# Reproductive Function During Chronodisruption: Model of Shiftwork in Rodents

**DOI:** 10.1210/endocr/bqaa151

**Published:** 2020-08-27

**Authors:** Valerie Leysen, Vincent Prevot

**Affiliations:** Univ. Lille, Inserm, CHU Lille, Lille Neuroscience & Cognition, Laboratory of Development and Plasticity of the Neuroendocrine Brain, Labex DistAlz, Lille, France

**Keywords:** Circadian Clock, phase-shift, GnRH, hypothalamus, preovulatory LH surge, fertility

The circadian clock is widely known to shape patterns in physiology, metabolism, and behavior. Disruptions of the circadian rhythm, such as disrupted light/dark cycles during shiftwork, are known to have adverse health effects, including on reproductive function. In women, this can take the form of menstrual irregularities, reduced fertility, and an increased risk of miscarriage (1). Despite these reports, the effect of chronodisruptive conditions on reproductive parameters in preclinical animal models remains largely unexplored.

Reproductive life in female mammals is characterized by the presence of regular cycles, namely the estrous cycle in rodents and the menstrual cycle in women. These cycles are tightly controlled by the hypothalamic–pituitary–gonadal (HPG) axis, a complex and tightly regulated feedback loop between hypothalamic gonadotropin-releasing hormone (GnRH) neurons, pituitary gonadotropins (luteinizing hormone [LH] and follicle-stimulating hormone [FSH]), and ovarian sex steroids. Together, these different components orchestrate the maturation of oocytes, which may be divided into 2 phases. The first phase, comprising metestrus–diestrus in rodents and the follicular phase in women, is marked by gradually increasing levels of FSH and the recruitment and development of ovarian follicles, leading up to a transient LH surge that triggers ovulation within a few hours in rodents and within 9 to 23 hours in women. This preovulatory surge in LH secretion marks the start of the second phase, comprising proestrus–estrus in rodents and the luteal phase in women, and takes place approximately every 4 to 5 days in rodents and every 28 days in women. This surge in LH, which occurs specifically around the transition from night to day or vice versa depending on species, suggests the involvement of the circadian clock, a possibility confirmed by the presence of severe impairments in estrous cyclicity and fertility after ablation of the suprachiasmatic nucleus (SCN), the master circadian clock, or by mutations in clock genes (see for review (1, 2)).

A report in the current issue of *Endocrinology* (3) by the Simonneaux laboratory demonstrates that chronodisruptive conditions have marked reproductive effects in female mice, in accordance with previous observations in women. By acutely shifting the light/dark cycle by 10 hours, the authors slightly increased the length of the estrous cycle and altered the occurrence and timing of the preovulatory LH surge almost immediately (as early as the first post-phase-shift cycle). This effect appeared to be transient and was attenuated rapidly by the third proestrus after the phase shift. Similar to the acute phase-shift protocol, the effect of a chronic phase-shift protocol (3 days on a 10-hour phase advance/4 days on a 10-hour phase delay over 9 months) on the length and regularity of the estrous cycle was limited. Nonetheless, chronic disruption severely affected the occurrence and timing of the preovulatory LH surge during the entire exposure period. In line with the observed alterations in the LH surge, female mice exposed to a chronic disruption of the light/dark cycle were less fertile, that is, showed fewer successful gestations and fewer pups per litter than control mice. One aspect that was not addressed in the study, however, is that of ovarian histology. Do chronodisrupted mice become hypogonadal? Do they develop polycystic ovary syndrome (PCOS)-like traits? This would indeed be an intriguing finding since, in addition to altered fertility, circadian misalignments cause adverse metabolic effects (4) that at least partially overlap those seen in women with PCOS (5).

The findings of this study raise several new questions, notably concerning the mechanisms by which the circadian clock could regulate reproductive parameters. The current view is that SCN signals act either directly on GnRH neurons or indirectly through preoptic region kisspeptin neurons that drive the preovulatory surge in LH secretion via vasoactive intestinal peptide (VIP) and vasopressin, respectively (2). It would be interesting to explore whether disruptions of the circadian rhythm alter VIP and vasopressin release in the rostral preoptic region and possibly alter the electrophysiological properties of GnRH and preoptic kisspeptin neurons. Equally interesting is the putative role of the ovarian circadian clock, which has also been suggested to alter the timing of ovulation (6), in this process. Could alterations in the preovulatory LH surge be attributed in their entirety to changes in the master SCN clock, or is there some interplay with peripheral circadian clocks?

It is also worth mentioning that chronodisruptive conditions could lead to accelerated ageing overall, including the premature ageing of the reproductive axis. A recent study suggests that premenopausal women working on rotating night shifts are at an increased risk of precocious menopause onset (7). In rodents, it has been reported that the timing and amplitude of the preovulatory LH surge are impaired starting from 12 months of age (8). In this context, it would be interesting to examine whether the observed impairments in the LH surge and fertility result from ageing-independent alterations (eg, an imbalance in VIP and vasopressin, the peptides secreted by SCN neurons) or whether exposure to a chronic shift protocol induces the accelerated ageing of the reproductive axis, which indirectly leads to these conditions.

A last point of interest lies in the long-term effects of chronodisruptive conditions. The authors observe that a chronic disruption of the light/dark cycle induces dramatic alterations in the profile of the preovulatory LH surge as early as the beginning of the phase shift protocol and lasting until just after it ceases (3). Reduced reproductive success is observed immediately after a 4-week disruption of the light/dark cycle. Notwithstanding the significant impact of these results, it would have been fascinating to see whether the alterations in reproductive parameters could be (partially) reversed following the experimental period by normalizing light/dark cycles, or whether these observations constitute lifelong reproductive alterations. This information would be particularly interesting for women working in long-term rotating shifts. It appears from this study that a brief disruption of the light/dark cycle, for example, an overseas journey, would not have a negative impact on women’s fertility. However, reproductive success appears to be adversely affected in women continuously exposed to circadian clock disruption, such as employees working rotating shifts, frequent travelers, and flight attendants. According to what has been described in the literature, the impact on reproductive capacity in the last group of women can present as menstrual irregularities, reduced fertility and increased risk of miscarriage (1). Yet, the fundamental question whether these alterations are completely reversible by following a normal light/dark cycle remains unanswered.

In conclusion, the study by Bahougne and colleagues demonstrating that exposure to chronic chronodisruptive conditions severely alters the preovulatory LH surge improves our understanding of how women working in shifts are more prone to reproductive dysfunction. Like many other studies focusing on relatively unexplored topics, these findings raise more questions than they answer regarding the mechanisms by which the circadian clock affects the reproductive axis and fertility. The way is clear for new and exciting studies that could help to further elucidate these mechanisms, and to increase the overall well-being of women working in long-term rotating shift work.

## Data Availability

Data sharing is not applicable to this article as no datasets were generated or analyzed during the current study.

## Abbreviations

FSH: follicle-stimulating hormone
GnRH: gonadotropin-releasing hormone
HPG: hypothalamic–pituitary–gonadal
LH: luteinizing hormone
PCOS: polycystic ovary syndrome
SCN: suprachiasmatic nucleus
VIP: vasoactive intestinal peptide

## Additional Information

***Disclosure Summary:*** The authors have nothing to disclose.
